# Integration of Real Signals Acquired Through External Sensors into RoboDK Simulation of Robotic Industrial Applications

**DOI:** 10.3390/s25051395

**Published:** 2025-02-25

**Authors:** Cozmin Cristoiu, Andrei Mario Ivan

**Affiliations:** Faculty of Industrial Engineering and Robotics, National University of Science and Technology Politehnica București, Splaiul Independentei No. 313, 060042 Bucharest, Romania; andrei_mario.ivan@upb.ro

**Keywords:** offline simulation, virtual commissioning, industrial robotics, RoboDK, sensors, analog signals, digital signals

## Abstract

Ensuring synchronization between real-world sensor data and industrial robotic simulations remains a critical challenge in digital twin and virtual commissioning applications. This study proposes an innovative method for integrating real sensor signals into RoboDK simulations, bridging the gap between virtual models and real-world dynamics. The proposed system utilizes an Arduino-based data acquisition module and a custom Python script to establish real-time communication between physical sensors and RoboDK’s simulation environment. Unlike traditional simulations that rely on predefined simulated signals or manually triggered virtual inputs, our approach enables dynamic real-time interactions based on live sensor data. The system supports both analog and digital signals and is validated through latency measurements, demonstrating an average end-to-end delay of 23.97 ms. These results confirm the feasibility of real sensor integration into RoboDK, making the system adaptable to various industrial applications. This framework provides a scalable foundation for researchers and engineers to develop enhanced simulation environments that more accurately reflect real industrial conditions.

## 1. Introduction

The integration of digital twin technology and virtual commissioning has become pivotal in advancing industrial automation, particularly within the framework of Industry 4.0. Digital twins serve as virtual counterparts of physical systems, enabling real-time monitoring, simulation, and optimization throughout a product’s lifecycle. This approach enhances predictive maintenance, operational efficiency, and system design [1]. Dedicated solutions are employed for programming, testing, and optimizing robotic tasks before integrating them into real production systems [2]. Software such as Process Simulate, Robotmaster, and RoboDK offer tools for simulating, testing, and optimizing industrial robotic processes, while also providing offline programming (OLP) functionality. Virtual commissioning allows for the testing and validation of control systems in a simulated environment before deployment, significantly reducing commissioning time and costs [3]. However, a significant challenge remains in accurately replicating dynamic interactions between robots and their environments, especially concerning real-time sensor data integration. Traditional simulation tools often rely on predefined or manually triggered signals, which may not capture the complexities of actual operational conditions [4].

Despite their powerful capabilities, these tools primarily simulate robotic behavior using predefined signals rather than real-time sensor data, limiting their ability to accurately reflect real-world conditions. Addressing this gap is crucial for developing simulations that closely mirror real-world scenarios, thereby improving the reliability and performance of automated systems. This study proposes an innovative approach to incorporating real-time sensor data into robotic simulations, bridging the divide between virtual models and physical operations.

Using simulation and offline programming software has become an important aspect of industrial robotic automation. Being one of the leading solutions on the market, RoboDK allows both integrators and researchers to simulate and study robotic industrial applications by providing a virtual 3D environment [5]. The software allows the kinematic modeling and integration of various robot types, along with analyzing a wide range of applications. However, these simulations lack the machine-to-machine dynamics that come with the data provided by the integrated sensors. These data are a very important aspect in all industrial robotic applications. Their management, which forms the station logic, is essential for the functionality and optimization of the application [6,7,8,9]. In the virtual environment provided by RoboDK (similar to other software solutions), these signals are simulated instead of being acquired from real sensors. Because the efficiency and relevance of a study developed using one of these software solutions depend on how capable that software is to emulate the real working environment and conditions, the simulation of the input signals represents a weak spot. The real sensors collect data from the working environment that has dynamic characteristics, especially when working with unstructured applications [10,11,12]. This leads to uncertainties that cannot be modeled in a virtual environment.

Another important aspect of using process simulation solutions is virtual commissioning. This is a method of testing and optimizing production systems that are developed in virtual environments before real-world integration. The essential element of virtual commissioning is testing the application logic, which refers to the management of I/O signals and the behavior of the integrated equipment with respect to those signals [13]. Because there are events in industrial applications that cannot be properly modeled in virtual environments, these must be emulated through certain elements and functions (such as “attach” or “duplicate object” options, which work differently from the real-world corresponding events) [14]. For these reasons, in most cases, the logic of the production system cannot be fully validated using the virtual environment alone. Most of these issues can be addressed by developing and using true digital twin models of real equipment. However, the digital twin concept has been developed mostly in recent years and has yet to be adopted on a large scale in the industrial field. On the other hand, even if a proper digital twin of the real system is used, virtual commissioning is still required in order to reduce the integration time of the system, and to check for errors in the control software. Thus, virtual commissioning, which is implemented by connecting the simulated system to a virtual or real PLC, is required to properly test the programming of the robotic industrial application [15].

There are certain applications that rely heavily on information acquired through external sensors [15]. For example, in assembly applications force/torque sensors are used to identify the gripping force of sensible parts or to identify the placing position of the components [16,17]. Collaborative applications also use various sensors to ensure safe interaction between robots and human operators [18]. While some robotic applications operate in structured environments with minimal sensor dependency, others require continuous real-time sensor data for accurate process execution. Digital twins and virtual commissioning play a crucial role in simulating such applications, yet most existing research relies on predefined or simulated signals rather than real-time sensor integration. The work performed in [2] emphasizes the importance of real-time process optimization in cyber–physical systems (CPS), while [3] classifies digital twin implementations and identifies real-time data synchronization as a major challenge. The effectiveness of virtual commissioning in reducing commissioning time and improving simulation accuracy is demonstrated in [4]. However, despite these advancements, most simulation tools still lack the capability to dynamically integrate real-world sensor data, limiting their ability to accurately model real-time industrial processes.

A significant challenge remains in bridging the gap between digital and physical systems by allowing real-time sensor-driven decision making in industrial robotic simulations. Many offline programming (OLP) software solutions, including Process Simulate, Robotmaster, and RoboDK, provide powerful tools for simulating, testing, and optimizing robotic operations but primarily rely on predefined non-dynamic input signals. This limitation reduces adaptability in scenarios where sensor feedback is critical for process validation. Addressing this shortcoming requires an approach that not only enhances sensor data integration but also maintains system flexibility, accuracy, and usability in an industrial setting.

This study introduces a software-driven framework for integrating real-time sensor data into RoboDK simulations, ensuring live sensor-driven robotic interactions. Unlike conventional simulation methods, which rely on predefined control sequences, our approach establishes a live communication link between real-world sensors and the virtual robotic environment. This is achieved through a custom Python-based interface, enabling real-time data exchange between external sensors and RoboDK software (version 5.7.4). By dynamically updating robotic behaviors based on real sensor inputs, the proposed method significantly improves simulation realism, adaptability, and predictive accuracy.

The core innovation of this study is the development of a customizable Python-based software interface that allows seamless sensor data transmission into RoboDK, eliminating the need for simulated approximations. The software is designed to be scalable, modular, and adaptable, supporting various industrial sensors and data acquisition methods. The use of Python scripting provides high flexibility for integrating additional features such as real-time data filtering, advanced control algorithms, and multi-sensor fusion techniques. Unlike previous approaches that rely on proprietary hardware or expensive industrial automation platforms, our solution remains cost-effective and open for further development.

To validate our approach, a framework was implemented and tested using external sensors interfaced with RoboDK via a Python-controlled communication pipeline. The integration allows for dynamic robotic interactions and enables process optimization in a more realistic and responsive digital twin environment. This contribution represents a significant step forward in making robotic simulations more representative of real-world industrial conditions, addressing a crucial gap in real-time digital twin applications for robotic automation.

## 2. State of the Art

**Figure 1:**
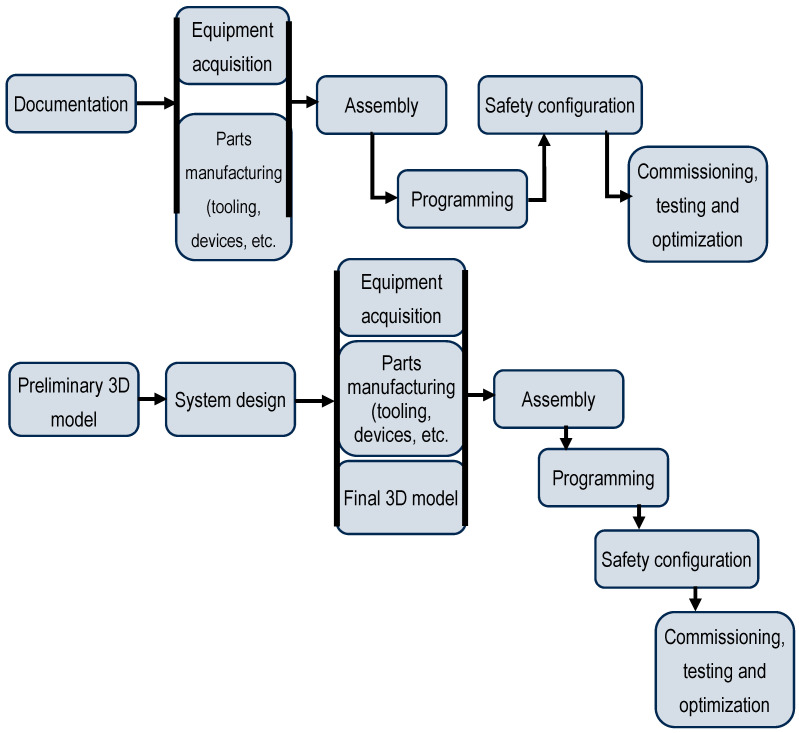
Implementation stages of a robotic industrial application: **up**—standard process design approach; **down**—virtual commissioning approach (as presented by Eguti et al. [19]).

The current industrial development status, with Industry 4.0 designation, is based on a hybrid approach to robotic application design. Eguti et al. [19] underline that the concept of virtual commissioning integrates aspects that are linked to digital manufacturing and simulation with hardware-in-the-loop elements. The research performs a comparative analysis between the conventional design of an automation system and the same design process based on a virtual commissioning approach. It is shown that the main objective of virtual commissioning is to reduce the time required for application design and integration, along with the product’s time-to-market. This is achieved through the possibility of simultaneously running several development stages, as shown in Figure 1.

However, various research works have shown that virtual commissioning has not yet been established as a standard procedure for automated application development [20,21,22,23]. While it is used on a large scale by big companies, smaller enterprises are not typically implementing the concept, mainly due to complexity and the high level of resources involved, including costs [24].

**Figure 2:**
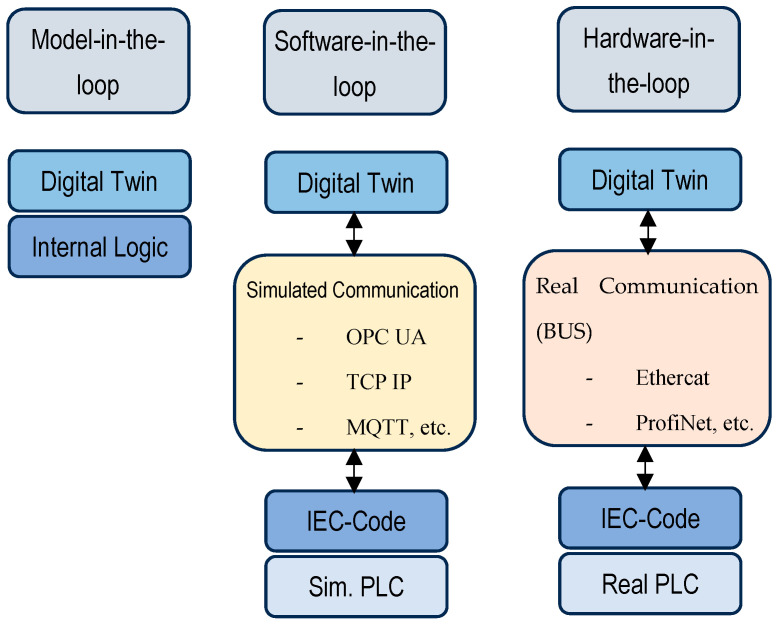
Software-in-the-loop and hardware-in-the-loop concepts, together with the model-in-the-loop approach, as presented by Ullrich et al. [29].

Virtual commissioning is, at its core, a testing method for application logic. In the literature, there are two approaches that are used: software-in-the-loop and hardware-in-the-loop [25,26,27,28]. The software-in-the-loop simulation tests the application programming (the station logic) together with the digital model/digital twin of the system, while using either a virtual or real PLC. The hardware-in-the-loop approach tests the real application programming running on a real PLC that communicates with the digital twin of the system through PROFINET, PROFIBUS, or other real communication standards [29]. Software-in-the-loop and hardware-in-the-loop concepts are presented in Figure 2.

Noga et al. [30] developed a concept of hybrid virtual commissioning. The study builds upon the concept of hardware-in-the-loop and aims to reduce the complexity and requirements of the simulation by using the real available equipment while simulating the rest of the system. Since one of the main advantages of simulation and virtual commissioning is the possibility of validating an application before purchasing any of the required equipment, a hybrid approach will be compatible with that argument, since only readily available equipment will be integrated with the simulation. This reduces the need to model those elements and ensures more accurate testing, since part of the setup is based on real components. The concept is validated using a real 3D vision camera and a SIMATIC ET 200SP I/O module connected to a real PLC. The camera scans the shape and position of objects placed in an unstructured configuration (random order and posture) and transfers the data into a virtual workspace. There, virtual commissioning for a pick-and-place application, including a delta robot, is performed.

## 3. System Design and Implementation

The proposed system enables real-time interaction between physical sensors and a virtual robotic simulation in RoboDK. This allows the digital twin environment to dynamically adjust based on live sensor input, improving the accuracy and adaptability of robotic simulations. The system architecture consists of three main components:

1. Sensor acquisition layer.
2. Simulation layer.
3. Python script.

To maintain clarity and conciseness, only the essential will be presented in the paper.

However, the entire project, including all files, virtual station, the full Python script and Arduino code, and detailed implementation instructions, is publicly available on GitHub: https://github.com/Cozmin90/rdk_signal_box01 (accessed on 10 January 2025).

### 3.1. Sensor Acquisition Layer

To interface these sensors with the simulation, an Arduino microcontroller is used as a sensor acquisition module. The choice of Arduino is based on its flexibility, low cost, and ease of integration. The microcontroller collects sensor data and transmits them to the PC via serial communication. The system is designed to accommodate both types of signals: digital and analog. For testing purposes only, two digital sensors are connected to the Arduino’s digital I/O pins (high/low states) and one analog sensor is connected to an analog I/O, providing values between 0 and 1023. The Arduino microcontroller and program continuously pool sensor values, processing them efficiently with debouncing and threshold filtering, and formatting them into structured messages that are sent via serial communication. Serial communication is initialized at 115,200 baud and, to prevent rapid fluctuations in digital sensor readings, a debouncing delay of 20 ms is added. The debouncing mechanism in the Arduino code prevents false readings caused by the mechanical nature of buttons, which tend to “bounce” when pressed or released. This is achieved by ensuring that each sensor state change is only registered if it remains stable for at least debounceDelay milliseconds. The millis() function is used to track the elapsed time since the last valid state change, filtering out unintended fluctuations. This enhances signal stability, ensuring that the Python (version 3.12.6) program only processes genuine button presses, preventing false triggers caused by mechanical noise. Also, to ensure that only significant analog value changes are transmitted, a threshold value of 20 units is added, thus reducing unnecessary data transmission. The sensor data are structured in a string as follows:

SENSOR_IDENTIFIER_VALUE_TIMESTAMP

Here are some examples of data output:

IO1_1_123450

IO2_0_123460

PD_350_123478

PD_372_123500

The numbers at the end of the strings represent the timestamps of each event, and their role is to be used by the Python script in order to compute the transmission delay.

**Figure 3:**
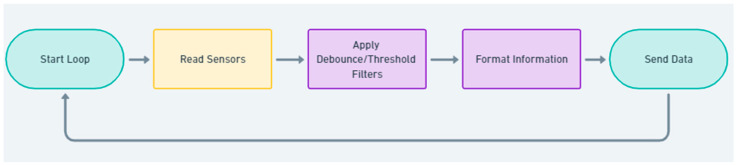
Arduino code logic.

The logical representation of the Arduino code is very simple, as follows in Figure 3:

### 3.2. Simulation Layer

The RoboDK software is used as a simulation tool for robotic applications. To perform a simulation in RoboDK, a station has to be created. The station represents the study of the robot application in a virtual environment. It represents a set of elements that includes virtual models for all physical components, target points and the corresponding robot paths, operations performed on the processed parts, coordinate systems, mechanisms and their kinematic configuration, tools, configuration files, programming elements, scripts, etc. These elements are organized in a logical structure; in most cases, in the form of hierarchical trees that are shown on the left side of the application window. For this study, two stations are created: one demo app (no real application) that is used only for demonstration and testing purposes and one station for a robotic palletizing application in order to demonstrate functionality in correspondence with a real case scenario.

#### 3.2.1. Case Study 1: Test Demo Application

The first station includes: two robots, target points and the corresponding paths, seven programming files that include robot movement instructions and instructions for signal management, together with the Python script. This virtual model for validation includes two articulated arm industrial robots. The models are the IRB 120 and the IRB 140 produced by ABB, each having six axes. A lamp is placed in front of each robot to provide visual feedback for digital signals. Also, to provide display data for the status of digital signals and for the value read by the photodiode (analog signal), a display is placed between the robots. The real photodiode (the common GM5539 model used in many Arduino projects) is wired to the analog input pin (A0) of the microcontroller. It is connected to the system through an analog-to-digital (ADC) convertor providing values between 0 and 1023 that are directly influenced by its changing resistance range: ~5 KΩ (bright light) to 200 KΩ (dark conditions). Being in the classroom, the photodiode is pointed at a screen that displays randomly changing images and colors.

**Figure 4:**
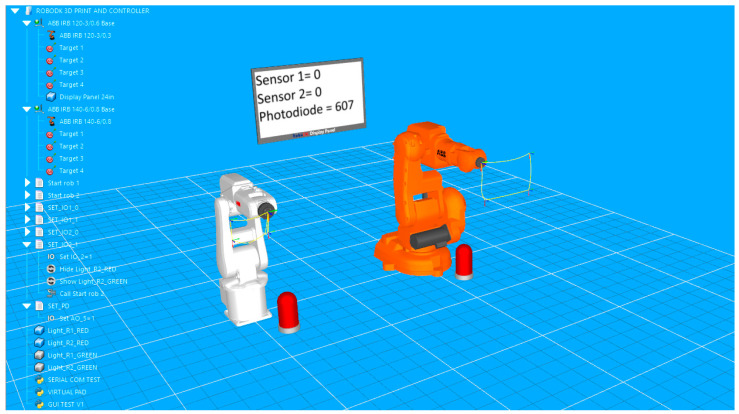
Validation simulation environment in RoboDK, including two industrial robots, two feedback lamps, a virtual display that shows real-time sensor readings, and the project tree, including objects, robot targets, and movement programs and Python scripts.

The setup of the application in RoboDK is displayed in Figure 4.

**Figure 5:**
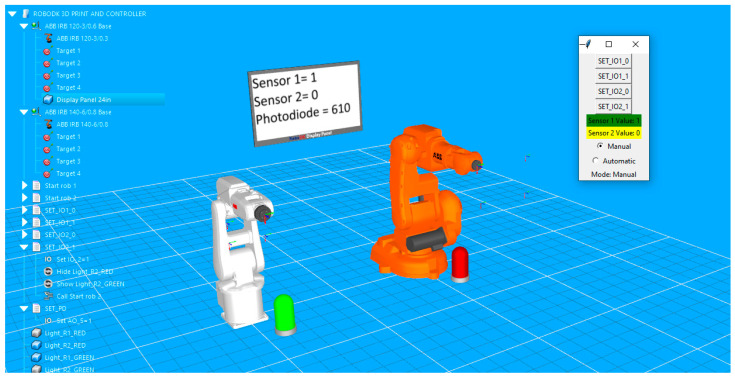
Visual feedback on the simulation environment in RoboDK at the state change of the first sensor: text and color feedback on the GUI, color feedback of the left-side lamp, and virtual display feedback.

For each robot, four target points are defined. The points are used to create an enclosed path roughly rectangular in shape. Both paths are simulated with a signal-based approach. The programming routines include instructions that wait for a certain change in I/O signals to trigger the robot’s movement in loop along the path. The simple graphic user allows the user to fast check the manual by turning the signals on or off if the lamp color changes corresponding to each of the two digital signals, if the correct state and values of the signals are displayed on the virtual display, and if the corresponding robot is executing its path following program. In Figure 5, signal IO_1 is set to high, so the lamp in front of the white robot turns green; on the display, its value turns to 1 and the robot has already moved to the next point on the path.

**Figure 6:**
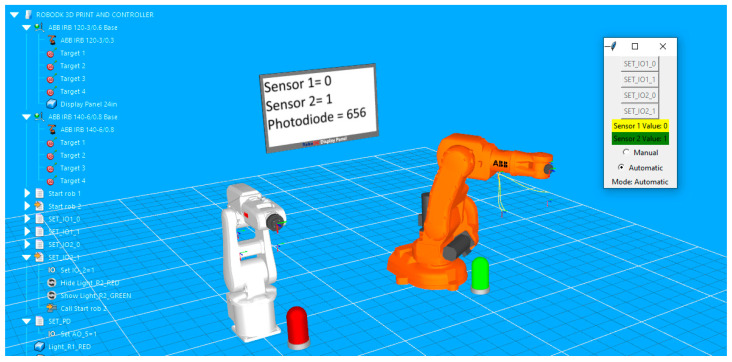
Image capturing the start of the movement routine of the orange robot triggered at the state change of the second sensor.

At the same time, the Python script responsible for receiving signal values from serial communication and transforming them into specific commands that trigger different actions in RoboDK virtual station is executed. The script is configured so that it can be loaded into the virtual station and launched at any time. It also implements a control panel in the application. The small GUI also allows the user, through a toggle button, to switch on “Automatic” mode. In this mode, digital signals cannot be changed from the GUI buttons (buttons become greyed out), but the actions in the stations are now triggered by the state of physical sensors (in this case, two push buttons). In Figure 6, the color of the second lamp indicates that the second button has been pressed, and the orange robot starts the execution of the movement program. 

**Figure 7:**
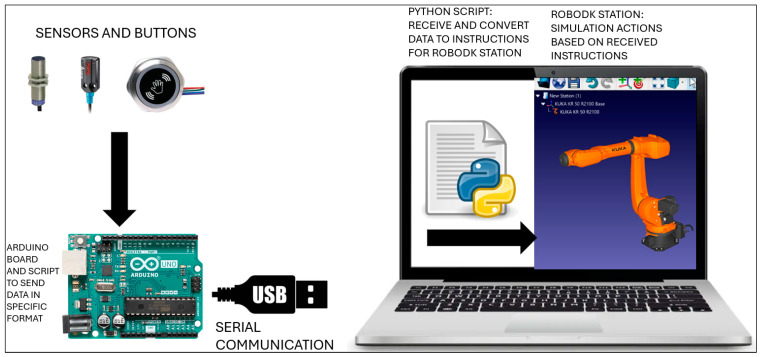
The hardware and software setup, including sensors and buttons, Arduino board, and a computer running RoboDK and the Python script.

In this case, based on the status of digital signals (received through serial communication from the push buttons connected to the Arduino module), one or both robots execute a task that is configured in the simulation environment. The signal can be associated with other events in the simulation, such as object hide/show, color change, modification of a variable value, executing a script, etc. For a real system, the change of an I/O signal can trigger events such as lighting an indicator, mechanism movement, activating a certain function of an equipment, program interrupts, etc. The data flow from sensors to the virtual system in RoboDK, going through the Arduino module, is illustrated in Figure 7.

The setup presented above is developed for testing. In this case, the analog sensor does not have any role and does not trigger any action in the simulation environment. Thus, a second simulation station is created in order to also use the analogic signal and to validate the concept in a useful and realistic application.

#### 3.2.2. Case Study 2: Robotic Palletizing Application

**Figure 8:**
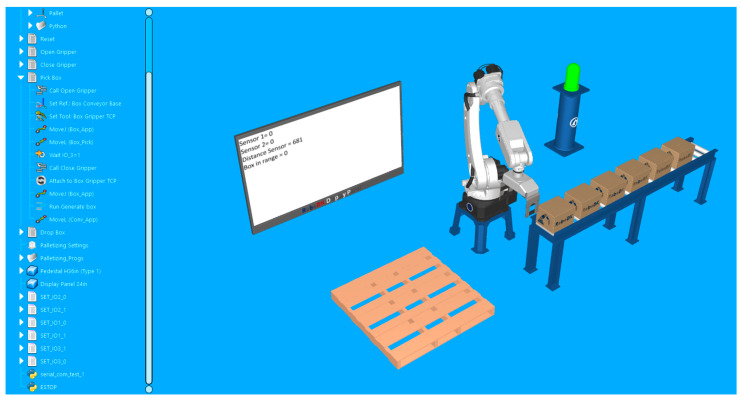
Validation simulation environment in RoboDK imitating a real-world automated palletizing operation. The robotic arm interacts with a conveyor system and a sensor-based feedback loop, displaying real-time distance and object detection data on a virtual screen.

For the second station, a simple robotic palletizing application is configured. For visual feedback, the lamp and the virtual display are kept in the station. In Figure 8, the layout can be observed.

The logic of the signals used in this station is the following:

- The first digital sensor signal is used to start the palletizing routine of the robot (it has the role of a start button).
- The second digital sensor signal is used to immediately interrupt the robot action (it has the role of an emergency stop button).
- The robot has to close its gripper and grab a box only when there is one exactly under it. So, the analog input signal is used to measure the distance from the gripper to the box and to trigger the grabbing action only when a box is in the corresponding range.

The correlation of signals is presented in Table 1.

This time, a HC-SR04 ultrasonic sensor is used to measure distances also connected directly to the Arduino board. This ultrasonic sensor model has one emitter and one receiver and can determine distance by measuring the time delay until the echo bounces from the object and returns to the receiver. To adjust the measured distance, we have to manually point it to some close objects in the classroom.

**Figure 9:**
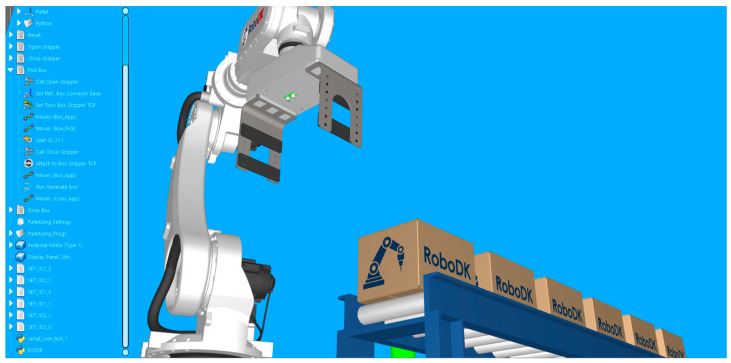
Placement of the virtual sensor on the gripper. The sensor is placed under the gripper in order to measure the distance to the box and close the gripper when the box is close enough.

In the virtual environment, the distance sensor is attached to the bottom of the robot gripper, as shown in Figure 9.

**Figure 10:**
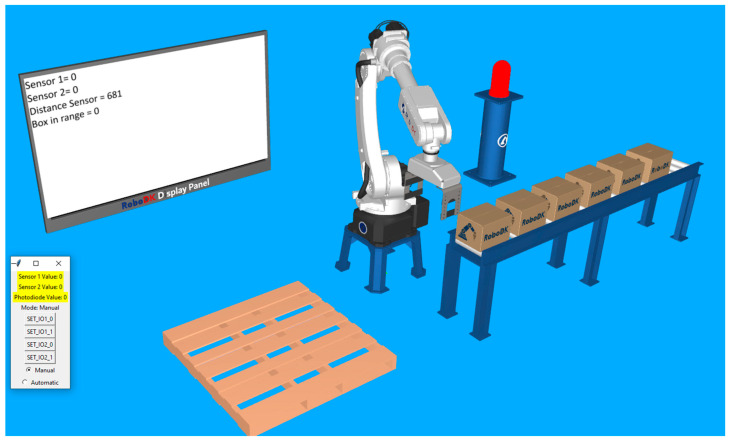
Station at rest with the robot waiting in its “home” position and the push of the first button (start button) to start the palletizing routine.

While the real start button is not pressed, everything is at rest. The lamp indicates the OFF status by the red color, both of the digital signals indicate the value zero on the display, the analog sensor displays its current value, and, since there is nothing close enough to the gripper, the “box in range” status is also zero. These elements can be observed in Figure 10.

**Figure 11:**
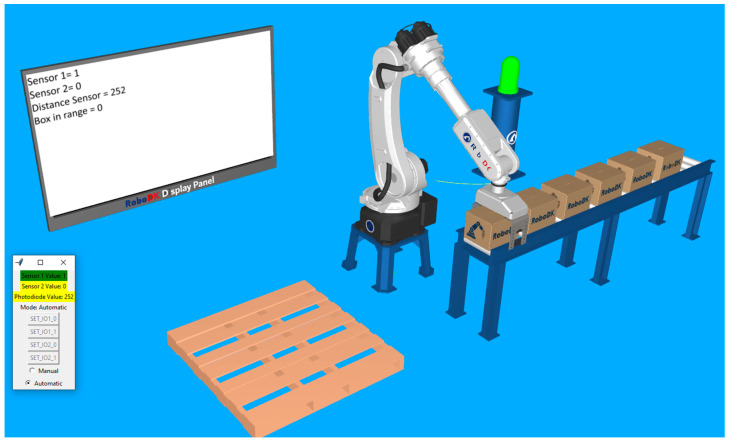
The robot approaches the first box until the distance value becomes smaller than the threshold and triggers the closing action of the clamps.

**Figure 12:**
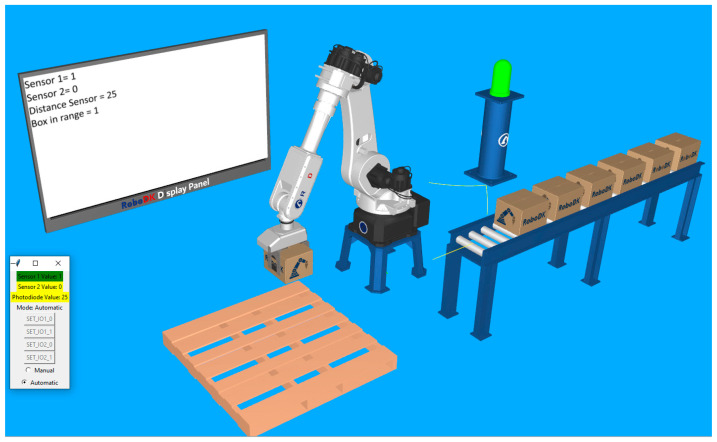
Measured distance reaches the threshold and triggers the “box in range” signal. The gripper clamps close and the robot continues its palletizing routine.

After the automatic mode is turned on and the start button has been pressed, the robot moves in the location determined to grab the boxes. The lamp becomes green, and the first digital sensor displays a value of 1. At this moment, the robot gripper is still open, but we can observe that, since the distance sensor has decreased, the box in range status is still 0. This is because it took a little time to put something in front of the real sensor and for it to detect and adjust the distance value. Right when the distance value drops under the specified threshold (value of 50), the robot closes its gripper, then moves the box approaching the palletizing location. These two sequences are presented in Figure 11 and Figure 12.

**Figure 13:**
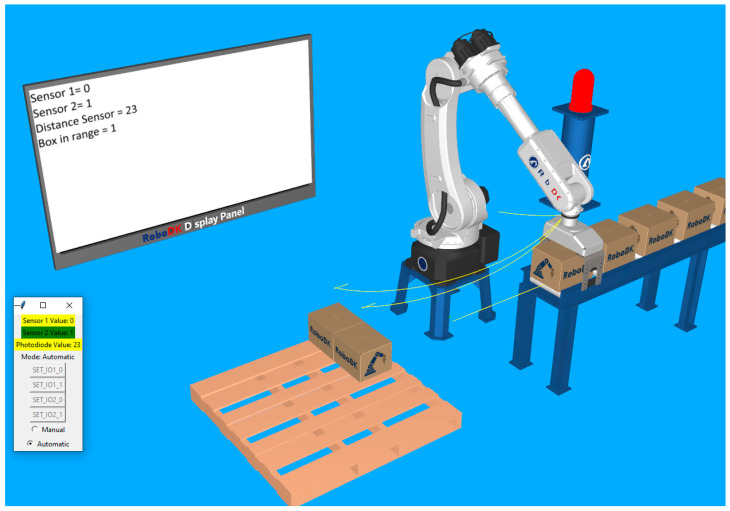
Program halts at the push of the second button (emergency stop). The status lamp becomes red, and the robot stops moving, even if the “box in range” signal is active.

To test the functionality of the emergency stop, the second button is pressed while the robot is in the position of grabbing another box, after confirmation of the “Box in range”. Every process immediately stops, and the visual indicators change accordingly. The moment of stopping the application at the signal of the second sensor is captured in Figure 13. 

The application described above is developed with the goal of providing a framework for testing the concepts approached in this study. It is deliberately kept as simple as possible to keep the testing results relevant for any robotic task, regardless of its particularity. The application has all the characteristics of a standard simulation, including real I/O signals, which are the core subject of this study. This study aims to address this issue and find a solution for integrating more realistic signals. For this purpose, a Python script is developed, and its functionality is detailed in the next section.

### 3.3. Python Script

The script acts as a connection between real sensors and the virtual signals included in the application, automatically calling the signal changing routines when necessary. The following libraries are included:

- Tkinter—for graphical interface/control panel elements.
- Serial—for receiving data from Arduino through serial communication.
- Threading—for starting a thread used for reading data received from Arduino.
- Robolink—for creating a link to RoboDK.

**Figure 14:**
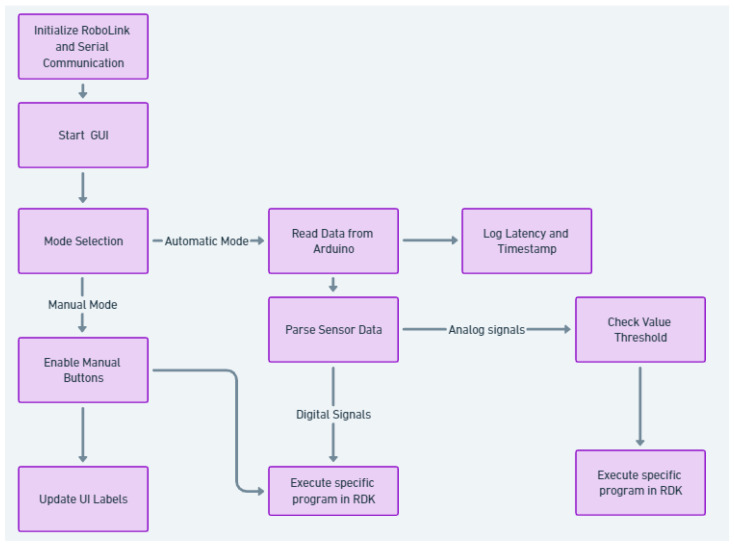
Logical diagram of the Python script.

The logical diagram of the script is illustrated in Figure 14.

**Figure 15:**
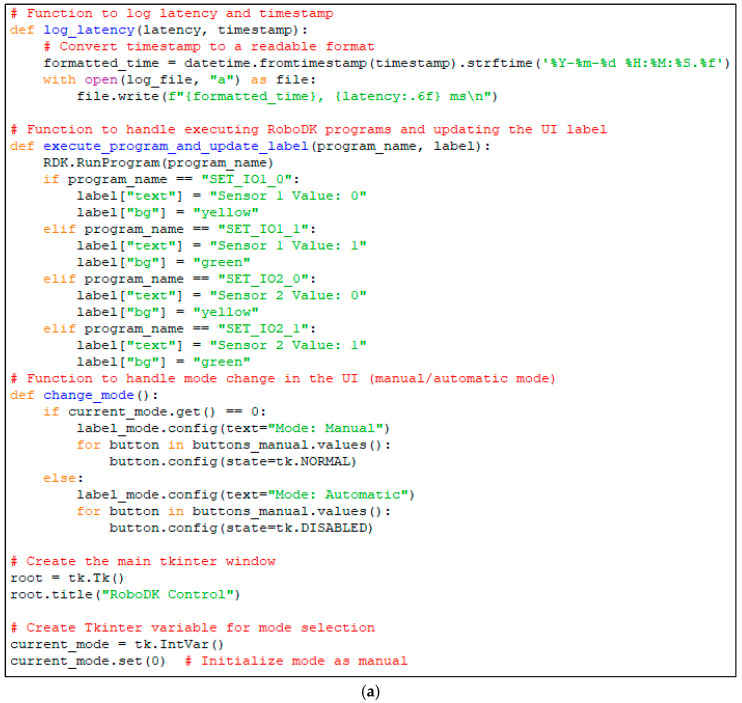

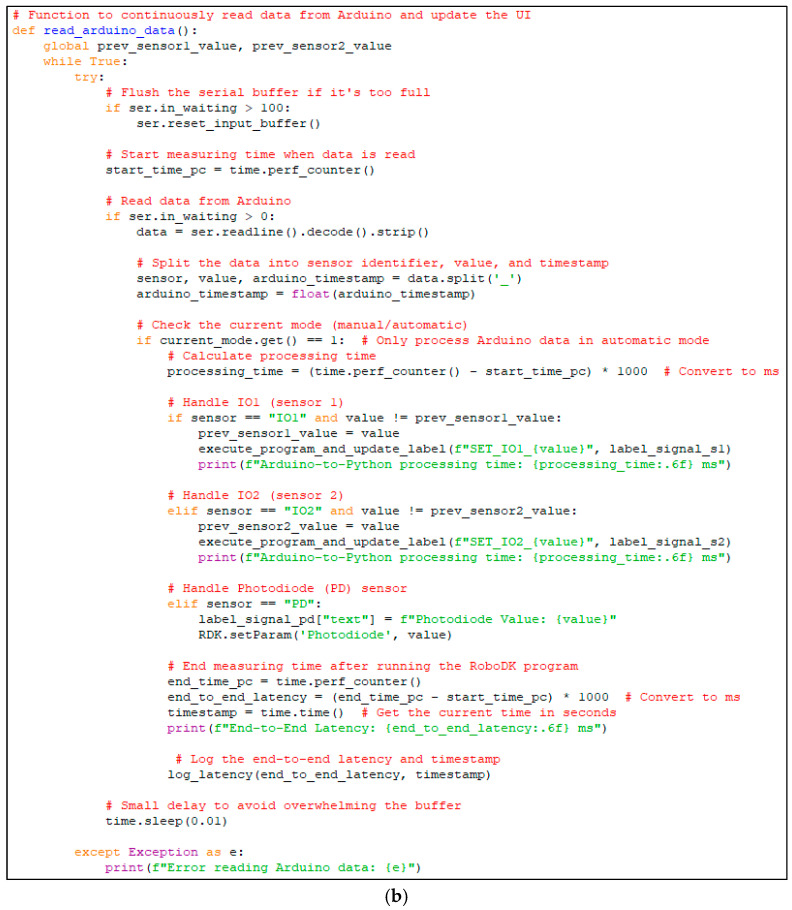
(**a**) Some Python script functions corresponding to the UI for selecting the operating mode (manual/automatic) and executing RoboDK programs in correspondence with received signal values. (**b**) Python script function responsible for continuously reading data from Arduino and triggering RoboDK actions based on received signals.

Serial communication is carried through the port to which the Arduino module is connected (in this case, Port 4) using an adjustable baud rate of 115,200 bits per second (as shown in Figure 15). In order to use the information configured in the graphical user interface and to process the data, the following functions are created.

- A function for changing signal values that also changes the color of the signal indicators (which show whether a signal is active or not).
- A function that reads the data received through serial communication from the Arduino board. These data are received in the form of character strings, such as “IO1_0”, “IO2_0”, “IO1_1”, “IO2_1”, and “PD_value”. These are associated with the buttons connected to the digital inputs of the Arduino board and with the analog sensor connected to one of the analog inputs. This function processes the data and then calls the required routines inside RoboDK or changes certain signal values in the simulation.
- A function that changes the signal management approach for the simulation. This function switches between manual and automatic mode. In manual mode, the signal values are changed through the push buttons provided by the control panel. In automatic mode, the signal values are determined by the data received through serial communication.

Some of these functions are also illustrated in Figure 15a,b, showing the modular configuration of the script.

The read_arduino_data function is responsible for receiving sensor signals from Arduino and processing them in real-time within the RoboDK simulation. To ensure that only the most recent data are processed, the serial buffer is flushed before reading new inputs. This prevents outdated sensor values from being used, particularly when switching between manual and automatic modes. Additionally, a debouncing mechanism is implemented at the Arduino level to prevent rapid unwanted fluctuations caused by mechanical button noise. This enhances the reliability of digital signal readings, ensuring stable interactions between the physical and virtual environments. Finally, the function also records end-to-end latency measurements, which are crucial for evaluating the real-time performance of the system. Key features in accordance with the code structure are presented in Figure 15b.

(a) The function continuously listens for incoming data from the Arduino and determines whether the received signal should trigger an action in the RoboDK simulation.
(b) Flushing the serial buffer (ser.reset_input_buffer()): ensures that old unread data do not cause delayed or outdated readings. Prevents buffer overflow when Arduino sends frequent sensor updates. Useful when switching between manual and automatic modes to avoid processing outdated data.
(c) End-to-End latency measurement: measures the time between when data are received from Arduino and when they trigger an event in RoboDK. Helps evaluate system responsiveness and optimize real-time performance.

The developed script has a simple structure, including 112 lines of code, together with the libraries mentioned above. Its purpose is to interact with the elements of a simulation configured in RoboDK, such as commands, routines, or events, and to link various external signals to these elements. Thus, the script allows the signals provided by external equipment and sensors to alter the elements of a simulation. In order to implement this concept, the following conditions are required:

- The station must be completely configured in RoboDK, including programs, modules, and routines. The various modules and routines should be readily available to be called when needed.
- The Python script must be integrated into the station. The Arduino module must be connected to the communication port defined in the script.
- The external sensors that are required in the application must be connected to the module. Also, the communication rate must be set to the same value both between the Arduino module and the computer and between the Arduino module and the Python script.
- The variable names that store the sensor values must be identical to the ones used for the name format in the Python script. For example, the “IO1_<value>” name format identifies the value of the IO1 signal. Thus, the variable used in the Arduino program that refers to the corresponding sensor signal must be named IO1. For the photodiode, the PD identifier is used. The “_<value>” component specifies the actual sensor value using integer data types. This naming format is important, as the script searches specifically for these elements. The received data take the form of a character string and the name of the signal together with its value are extracted based on the location of the “_” character.
- The monitoring of the serial communication (serial monitor) from Arduino IDE must be closed, so that it does not interfere with the communication between the module and the script.

## 4. Results and Discussions

The primary objective of this study is to develop a practical and user-friendly solution that enables the integration of real-world signals into RoboDK simulations in a simple and efficient manner. The result is a compact, well-structured, and easy-to-implement script that introduces a crucial functionality into a simulation environment that previously lacked this capability. Our approach is not merely theoretical but is aimed at developing a method that can be immediately applied in real-world scenarios, providing researchers and engineers with a flexible and adaptable tool for testing industrial processes based on real sensor data. This study thus demonstrates the feasibility and practical utility of the proposed solution, contributing to the expansion of simulation and optimization capabilities in industrial applications. Ultimately, the most important and only relevant aspect when evaluating the efficiency and performance of the developed solution is the measurement of system latency. While the implementation is compact, easy to use, and introduces a crucial functionality into the simulation environment, the true value of this method is validated solely by measuring its reaction time. These measurements are essential to demonstrate that the system can operate under conditions close to reality, ensuring a sufficiently fast response for the targeted industrial applications. Based on this, we conduct a series of rigorous tests to assess the total system latency.

Latency time of the interface is defined as the time that passes between transmitting the data from the Arduino board and the execution of the code sequence triggered by the corresponding signal in RoboDK. This time period has two main elements: the time that reflects the communication speed between Arduino and the computer, and the time required for the triggered actions to be executed in RoboDK. This total time is called end-to-end latency. It reflects the capacity of the system—formed by the Arduino board, the Python script, and RoboDK—to complete certain actions based on sensor inputs. This duration can be influenced by various factors, such as the configured communication speed (baud rate), microprocessor specifications, algorithm efficiency, etc. Considering that the Arduino board used is the UNO model, the baud rate is set to the maximum value of 115,200.

**Figure 16:**
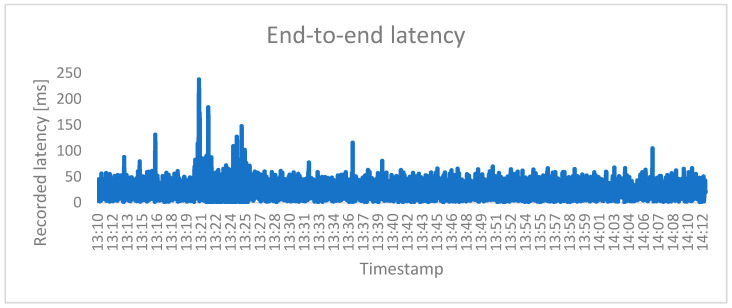
Graphical representation of the latency evolution during 60 min of continuous operation.

To evaluate the response of the system, the application runs for 60 min. The timestamp and end-to-end latency are recorded at each signal value change. The latency_log.txt file can be found in the repos, along with the other relevant files. The total recording count is 4126. By calculating the average of these times, a medium value of 23.97 ms is determined. Figure 16 illustrates the latency evolution for the measurement time of 60 min.

**Figure 17:**
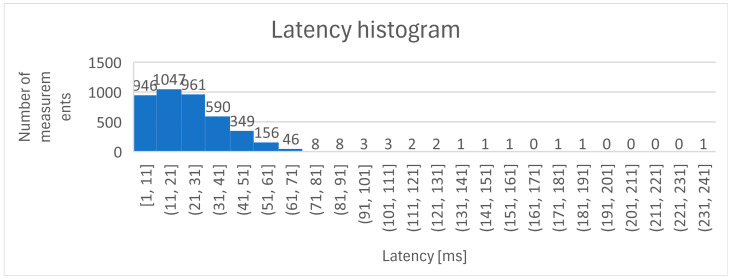
Histogram of latency measurements during test period of 60 min.

Looking at the graphical representation of the latency evolution, the communication delay may sometimes look like a random event. The reason is that the analog sensor is placed in front of a screen that continuously and randomly displays a changing image. It is possible that the transition between colors and images is not always so sudden that it quickly exceeds the threshold so that the sensor detects the change and sends the signal. The delay distribution is presented in Figure 17.

While the proposed framework successfully demonstrates the feasibility of real-time sensor integration into RoboDK simulations, certain limitations should be acknowledged.

(a) Hardware Dependency:
  - The current implementation relies on an Arduino-based platform for sensor acquisition, which may not be representative of higher-end industrial PLCs or edge computing systems.
  - While the approach is modular, testing with more advanced industrial controllers could provide additional validation.
(b) Communication Latency and Real-Time Constraints:
  - The system achieves an average latency of 23.97 ms, which is acceptable for most robotic applications but may not be sufficient for high-speed industrial processes requiring near-zero latency.
  - Future improvements could explore faster communication protocols (e.g., TCP/IP, UDP, or real-time fieldbuses like EtherCAT) to reduce response time.
(c) Limited Sensor Modalities Tested:
  - Future work should explore multi-sensor fusion techniques to enhance simulation realism and decision-making capabilities.
(d) Simulation vs. Real-World Performance:
  - While real sensor data are used, RoboDK remains a simulation environment, and the actual performance of the system in a physical industrial setting has not been tested.
  - Future work should focus on deploying the proposed approach in a real industrial setup, comparing simulated vs. real execution outcomes.
(e) Scalability and Complex Systems:
  - The current study demonstrates a single robot and a limited number of sensors. Expanding this to multi-robot systems with complex sensor networks introduces additional challenges, such as data synchronization, computational load, and system architecture complexity.

By recognizing these limitations, this study sets the foundation for future enhancements, ensuring that the proposed approach remains adaptable to industrial automation trends and evolving robotic systems.

The direct integration of robotic systems without prior virtual commissioning and real-signal-based simulation poses significant operational risks, such as unexpected failures, prolonged downtime, and suboptimal system performance [31,32]. To mitigate these challenges, the proposed real-time robotic simulation framework enhances sustainability in industrial automation by minimizing material waste, optimizing production efficiency, and enabling precise virtual commissioning. This approach accelerates deployment time, and saves energy and potential material lost by identifying and addressing potential issues before physical implementation [33].

## 5. Conclusions

This study presents the development of an innovative and practical framework for integrating real-world sensor signals into industrial robotic simulations. Unlike conventional simulation environments that rely on predefined or artificially generated signals, the proposed system enables real-time sensor-driven interactions within RoboDK. This is achieved through a lightweight and efficient Python-based interface that links an Arduino-powered hardware module to the simulation environment. The Arduino board is responsible for acquiring sensor data, which are then transmitted via serial communication and processed by the Python script, effectively translating physical sensor states into virtual simulation triggers.

The proposed system distinguishes itself from existing solutions by offering a highly practical, adaptable, and efficient approach to real-time robotic simulation enhancements. Its main characteristics include ease of implementation and use, as the framework is designed to be quickly and seamlessly integrated into any RoboDK application without requiring significant modifications. It has minimal resource requirements, being a lightweight solution consisting of only 112 lines of code, occupying just 7 KB of memory, and requiring negligible computational power. Additionally, the system ensures high responsiveness and low latency, with a measured total latency of 23.97 ms, making it suitable for real-time industrial applications. By incorporating real sensor feedback, the method enhances the realism of digital twins, ensuring that environmental dynamics and data variations are accurately represented, thus improving simulation reliability. Moreover, the approach supports a broad range of sensors, and its modular architecture allows for easy expansion to accommodate multiple input sources and various sensor types, making it highly flexible and scalable.

Compared with existing studies that focus on static simulations, the proposed approach introduces a real-time data-driven methodology for robotic process validation. Traditional offline programming (OLP) solutions and similar platforms lack native support for real-time external sensor integration. This study fills that gap by demonstrating a working prototype that effectively bridges the virtual and physical domains. The measured low-latency performance confirms that the system is not just a theoretical concept but a practical and deployable solution for industrial robotic research and automation.

The current implementation lays the foundation for further advancements in sensor-driven robotic simulations. Future work will focus on developing a dedicated microcontroller-based module to replace the current Arduino-based solution, improving hardware compatibility and integration with industrial controllers. Additionally, efforts will be made to enhance RoboDK integration by transitioning from a Python script to a dedicated RoboDK plugin, offering a graphical interface for real-time sensor interaction. Another key improvement will be the implementation of real-time sensor calibration, allowing users to define analog reference values, ensuring compatibility with a wider variety of industrial sensors. Finally, alternative communication protocols, such as UDP, MQTT, or real-time fieldbuses (e.g., EtherCAT, PROFINET), will be explored to further reduce latency and improve synchronization with industrial automation systems.

This research represents a significant advancement in the domain of real-time digital twins and virtual commissioning, enabling more precise, responsive, and adaptable industrial robotic simulations.

## Figures and Tables

**Table 1:** Signal correlation.

I/O Signal	Sensor Type	Description	Mapped RDK Action
IO1	digital	real push button	start robot movement routine
IO2	digital	real push button	stop every process (E-stop)
Distance	analog	real distance sensor	toggle IO3 on or off
IO3	digital	simulated digital signal	close gripper

## Data Availability

To promote further use and development of this framework, the entire project has been made available as open source. All the files are available at: https://github.com/Cozmin90/rdk_signal_box01.
